# Effects of crystallization and dopant concentration on the emission behavior of TiO_2_:Eu nanophosphors

**DOI:** 10.1186/1556-276X-7-1

**Published:** 2012-01-03

**Authors:** Mou Pal, Umapada Pal, Justo Miguel Gracia Y Jiménez, Felipe Pérez-Rodríguez

**Affiliations:** 1 Instituto de Física, Benemérita Universidad Autónoma de Puebla, Apartado Postal J48, Puebla, Pue., 72570, México

**Keywords:** titania nanoparticles, europium doping, optical properties, photoluminescence

## Abstract

Uniform, spherical-shaped TiO_2_:Eu nanoparticles with different doping concentrations have been synthesized through controlled hydrolysis of titanium tetrabutoxide under appropriate pH and temperature in the presence of EuCl_3_·6H_2_O. Through air annealing at 500°C for 2 h, the amorphous, as-grown nanoparticles could be converted to a pure anatase phase. The morphology, structural, and optical properties of the annealed nanostructures were studied using X-ray diffraction, scanning electron microscopy, energy-dispersive X-ray spectroscopy [EDS], and UV-Visible diffuse reflectance spectroscopy techniques. Optoelectronic behaviors of the nanostructures were studied using micro-Raman and photoluminescence [PL] spectroscopies at room temperature. EDS results confirmed a systematic increase of Eu content in the as-prepared samples with the increase of nominal europium content in the reaction solution. With the increasing dopant concentration, crystallinity and crystallite size of the titania particles decreased gradually. Incorporation of europium in the titania particles induced a structural deformation and a blueshift of their absorption edge. While the room-temperature PL emission of the as-grown samples is dominated by the ^5^D_0 _- ^7^F*_j _*transition of Eu^+3 ^ions, the emission intensity reduced drastically after thermal annealing due to outwards segregation of dopant ions.

## Introduction

Luminescent nanomaterials have gained considerable attention in recent years due to the breakthrough developments of technology in various areas such as electronics [1,2], photonics [3], displays [4,5], optical amplifications [6], lasers [7], fluorescent sensing [8], biomedical engineering, [9] and environmental control [10]. The long emission lifetime and rich spectral properties of certain rare-earth [RE] ions are highly attractive in many ways. However, RE ions alone are weakly fluorescent due to the parity forbidden f-f transitions [11]. Therefore, the use of host materials is crucial to excite the RE ions efficiently in a wide spectral range in order to utilize their full potential in optoelectronic devices. Oxide lattices have proved to be an excellent host material due to their good thermal, chemical, and mechanical stabilities [12,13]. Among them, Y_2_O_3 _is a promising host for RE ions due to its low phonon frequencies, which make the nonradiative relaxation of the excited states inefficient [14]. However, the high costs associated with synthesis have restricted its further use. As an alternative, TiO_2_, a well-known wide bandgap semiconductor, has demonstrated the possibility to be a good sensitizer to absorb light and transfer energy to RE ions. Moreover, the high refractive index and high transparency of TiO_2 _in the visible and infrared regions make it possible to use in optical devices. The additional advantages of using TiO_2 _are its low fabrication cost and good thermal and mechanical stabilities. However, due to the large mismatch of ionic radii (Eu^+3 ^= 0.95 Å and Ti^+4 ^= 0 0.68 Å) and charge imbalance between the Ti^+4 ^and Eu^+3 ^ions, successful incorporation of Eu ions into TiO_2 _nanocrystals through a soft, wet-chemical route still remains a great challenge. In most of the cases, Eu^+3 ^ions either tend to locate on a crystal surface, causing an undesired Eu-Eu interaction, or form Eu_2_O_3 _aggregates, which act as quenching sites, resulting in a drastic decrease in the luminescent intensity [15]. Numerous studies have been realized on the synthesis and optical characterization of Eu^+3^-doped TiO_2 _with the objective of improving the luminescence of the Eu^+3 ^ions by energy transfer from TiO_2_. It has been reported that the mesoporous, semicrystalline TiO_2 _films are ideal matrices for incorporating Eu^+3 ^ions in which the sensitized photoluminescence [PL] emission is due to the energy transfer from the TiO_2 _to Eu^+3 ^ions in an amorphous TiO_2 _region [16]. However, the emission intensity of Eu-doped TiO_2 _nanostructures has been found to reduce greatly or even disappear completely after annealing at high temperatures [17]. In the literature, we can find several explanations for this behavior such as phase transition [18], segregation of Eu_2_O_3 _from TiO_2 _[19], or formation of a highly symmetric structure of Eu_2_Ti_2_O_7 _at high temperatures [20]. Therefore, the fabrication of structurally pure, concentration-controlled, single-phase TiO_2_:Eu nanostructures with a controlled emission behavior is still a challenging task for their utilization in optoelectronics.

For the application in luminescent devices, small phosphor particles of a spherical morphology, narrow size distribution, and low dispersity are desired to improve their emission intensity and screen packing [21]. To meet these demands, a variety of synthesis methods have been applied to fabricate RE-doped titania nanoparticles. Luo et al. could prepare Eu-doped TiO_2 _nanodots in the 50- to 70-nm size range by a phase-separation-induced self-assembly method [15]. Yin et al. have studied the luminescence properties of spherical mesoporous Eu-doped TiO_2 _particles of 250 nm in diameter obtained through a nonionic surfactant-assisted soft chemistry method [16]. Ningthoujam et al. could obtain Eu^+3^-doped TiO_2 _nanoparticles by urea hydrolysis in an ethylene glycol medium at a temperature of 150°C [17]. Chi et al. have synthesized Eu-doped TiO_2 _nanotubes by a two-step hydrothermal treatment [22]. On the other hand, Julian et al. could synthesize Eu^+3^-doped nanocrystalline TiO_2 _and ZrO_2 _by a one-pot sol-gel technique [23].

In the present work, we report the incorporation of Eu^+3 ^ions in TiO_2 _nanoparticles by a simple and versatile sol-gel technique which could be extended to different lanthanide and transition metal ions in order to obtain multifunctional materials. The particles thus obtained have shown a perfectly spherical shape, improved size distribution, and excellent luminescent characteristics, elucidating the possibility of applying RE-doped titania nanoparticles as an efficient luminescent material. The dependence of the PL intensity of the nanophosphors on doping concentration and thermal annealing has been discussed.

### Experimental details

Eu-doped TiO_2 _nanoparticles were prepared according to the following procedures: 2.5 ml of titanium tetrabutoxide (97%, Aldrich) was added slowly to 25 ml of anhydrous ethanol inside a glove box under nitrogen atmosphere and kept under magnetic stirring for 1 h at room temperature. Hydrolysis of the mixture was carried out by dropwise addition into 50 ml of deionized water inside a round-bottom flask under vigorous stirring. Prior to the addition, the pH of the water was adjusted to 3.0 by adding a nitric acid (0.1 M) solution in order to avoid the formation of europium hydroxide. The temperature of the mixture was maintained at 4°C to retard the hydrolysis rate.

Eu(III)-doped samples were prepared following the same procedure but dissolving the required amounts of Eu(NO_3_)_2_·6H_2_O corresponding to 0.5, 1, 2.5, and 5 mol% (nominal) in water before the addition of the Ti precursor. The white precipitate of TiO_2 _was separated through centrifugation, washed several times with water and ethanol, and finally dried at room temperature to obtain resulting materials. In order to induce crystallization, the as-grown samples (both the undoped and Eu-doped) were thermally treated at 500°C for 2 h in air atmosphere.

The crystalline phase of the nanoparticles was analyzed by X-ray diffraction [XRD] using a Bruker D8 DISCOVER X-ray diffractometer with a CuKα radiation (*λ *= 1.5406 Å) source. The size, morphology, and chemical composition of the nanostructures were examined in a JEOL JSM-6610LV field-emission scanning electron microscope [FE-SEM] with a Thermo Noran Super Dry II analytical system attached. The absorption characteristics of the synthesized samples in a UV-Visible [UV-Vis] spectral range were studied by diffuse reflectance spectroscopy (Varian Cary 500 UV-Vis spectrophotometer with DRA-CA-30I diffuse reflectance accessory). Micro-Raman spectra of the powder samples were acquired using an integrated micro-Raman system. The system includes a microspectrometer HORIBA Jobin Yvon HR800, an OLYMPUS BX41 microscope, and a thermoelectrically cooled CCD detector. The 332.6-nm emission of a He-Ne laser was used as the excitation source. PL measurements were performed at room temperature using a Jobin Yvon iHR320 spectrometer (HORIBA) with a 374-nm emitting diode laser as an excitation source.

## Results and discussion

**Figure 1:**
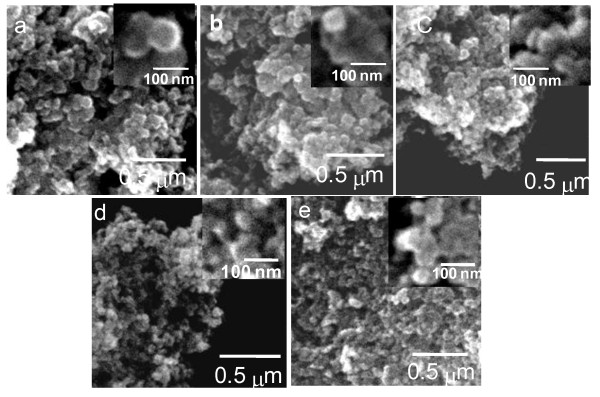
**Typical SEM images**. (**a**) Undoped, (**b**) 0.5%, (**c**) 1.0%, **(d**) 2.5%, and (**e**) 5.0% (nominal) Eu-doped TiO_2 _nanoparticles. The insets show magnified images of some particles for each sample.

**Figure 2:**
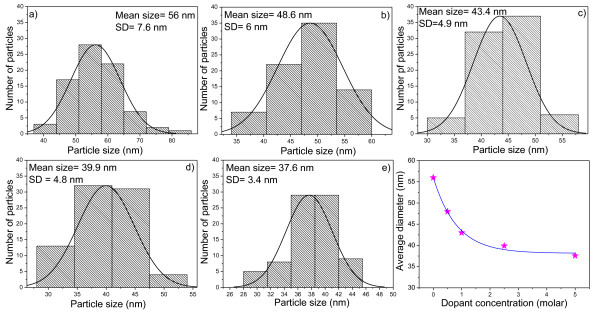
**The size distribution histograms and corresponding Gaussian fits**. (**a**) 0.0%, (**b**) 0.5%, (**c**) 1.0%, (**d**) 2.5% and (**e**) 5.0% (nominal) of the Eu dopant. Variation of the particle size with dopant concentration is shown in the bottom right. The average diameter decreased exponentially with the increasing molar concentration of Eu^+3 ^ions.

Figure 1 shows the SEM images of undoped and doped TiO_2 _nanoparticles revealing their general morphology. The corresponding size distribution histograms and the variation of average size with dopant concentration are presented in Figure 2. Formation of titania nanoparticles of a spherical morphology and narrow size distribution can be seen from the SEM micrographs. Compared with the undoped TiO_2_, the average size of the Eu-doped TiO_2 _nanoparticles decreases almost exponentially with the increase of the dopant concentration, suggesting that the incorporation of Eu ions suppresses the growth of TiO_2 _nanocrystals to a great extent.

**Figure 3:**
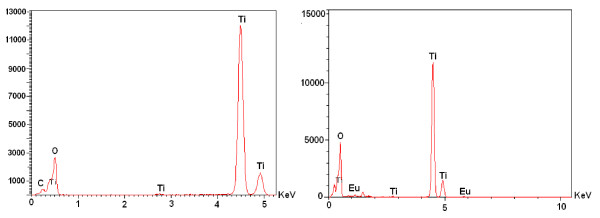
**EDS spectra of the undoped and 5.0 mol% (nominal) Eu-doped TiO_2 _samples**.

In order to verify the presence of Eu in the doped samples, they were analyzed by energy-dispersive spectroscopy [EDS]. EDS spectra and estimated composition of the samples are presented in Figure 3 and Table 1, respectively. The EDS spectra clearly revealed that the emission peaks correspond to O, Ti, and Eu, along with the carbon peak which might have come from the carbon tape used to fix the samples on the sample holder. A systematic decrease in the content of titanium and an increase in the relative content of europium are observed with the increasing nominal concentration of the dopant in the samples.

**Table 1:** EDS estimated quantitative composition analysis of undoped and Eu-doped TiO_2 _nanoparticles

Nominal Eu concentration in the sample (mol%)	Oxygen (atom %)	Titanium (atom %)	Europium (atom %)
0.0	64.73	35.27	0.0
0.5	65.10	34.63	0.27
1.0	66.42	33.23	0.35
2.5	66.59	32.84	0.57
5.0	67.76	31.30	0.94

**Figure 4:**
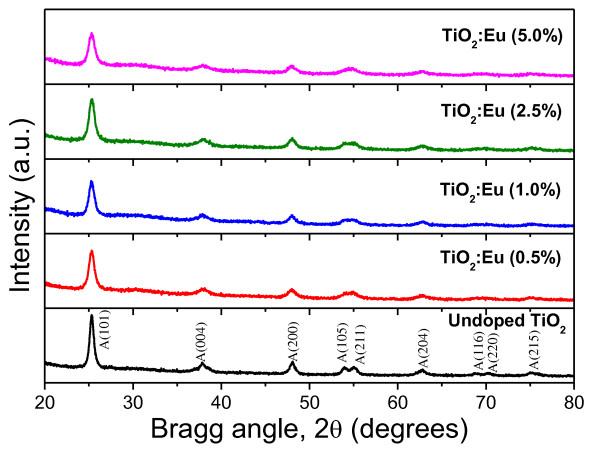
**XRD patterns of the Eu-doped TiO_2 _nanoparticles showing their pure anatase phase**.

The XRD patterns of the undoped and Eu-doped phosphor particles (Figure 4) revealed the presence of TiO_2 _exclusively in an anatase (tetragonal) phase (JCPDS 84-1286) after thermal annealing. In general, the intensity of the diffraction peaks decreases greatly with the increase of doping concentration, indicating a loss of crystallinity due to lattice distortion. When Eu^+3 ^ions are incorporated into the periodic crystal lattice of TiO_2_, a strain is induced into the system, resulting in the alteration of the lattice periodicity and decresae in crystal symmetry. As can be seen from the XRD patterns, the diffraction peaks get broadened as the Eu^+3 ^concentration is increased, suggesting a systematic decrease in the grain size. The peaks which correspond to the crystal planes (101) and (200) of the anatase phase are selected to calculate the lattice parameters of the undoped and Eu-doped TiO_2 _nanocrystals. Using the relations *d*_(hk1)_= *λ*/2 sin*θ *(Bragg's law) *and $d_{\left(\text{hkl}\right)}=\;{\left(h^{2}∕a^{2}+\;k^{2}∕a^{2}+\;l^{2}∕c^{2}\right)}^{-1∕2}$*, the lattice parameter and unit cell volume of the samples were evaluated (Table 2). Here, hkl are the Miller indices; *a, b*, and *c *are the lattice parameters (in a tetragonal system, *a = b ≠ c*); *d*_(hkl) _is the interplanar spacing between the crystal planes (hkl); *λ *is the X-ray wavelength; and *θ *is the diffraction angle. As can be seen from the estimated data, the estimated lattice parameters and unit cell volume values for the doped TiO_2 _nanoparticles deviate considerably from those of the undoped sample due to the incorporation of Eu^+3 ^ions into the TiO_2 _lattice, which induces the local distortion of the crystal structure.

**Table 2:** Lattice parameters and cell volume of different samples calculated from XRD results

Sample	*a *(Å)	*c *(Å)	Cell volume (Å^3^)
TiO_2_:Eu 0%	3.7830	9.5346	136.4505
TiO_2_:Eu 0.5%	3.7945	9.5379	137.3288
TiO_2_:Eu 1.0%	3.7864	9.5476	136.8827
TiO_2_:Eu 2.5%	3.7851	9.6175	137.7897
TiO_2_:Eu 5.0%	3.7863	9.5723	137.2291

**Figure 5:**
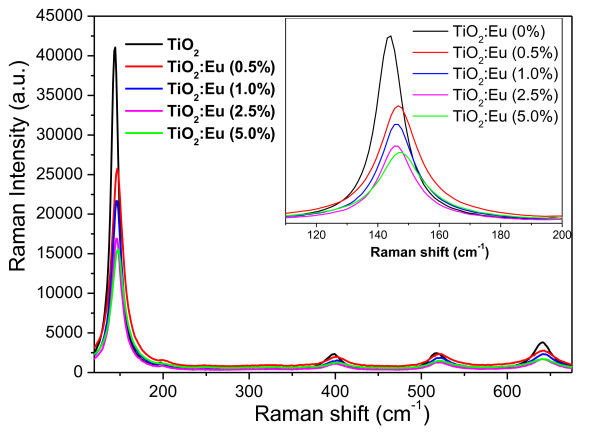
**Raman spectra of the undoped and Eu-doped TiO_2 _nanoparticles**. Peak broadening and red shift of the Raman-active mode at 144 cm^-1 ^on the increasing dopant content are shown as inset.

Micro-Raman spectroscopy is a powerful tool to investigate the structural properties of nanostructures, monitoring the unusual band broadening and shifts of Raman bands associated with particle size. According to the Heisenberg uncertainty principle, the particle size and phonon position hold the following relationship:

(1) $$\Delta X\;\Delta P\;\geq \hbar^{2}∕4,$$

where *ΔX *is the particle size, *ΔP *is the phonon momentum distribution, and *ħ *is the reduced Planck's constant. As the particle size decreases, the phonon is increasingly confined within the particle, and the phonon momentum distribution increases. This situation leads to a broadening of the momentum of the scattered phonon according to the law of conservation of momentum, causing a peak broadening as well as a shift of the Raman bands [24]. Figure 5 shows the Raman spectra of the undoped and Eu-doped TiO_2 _nanoparticles. According to group theory, anatase has six Raman-active modes (A_1g _+ 2B_1g _+ 3E_g_) [25]. Ohsaka reported the Raman spectrum of an anatase single crystal where six allowed modes appeared at 144 (E_g_), 197 (E_g_), 399 (B_1g_), 513 (A_1g_), 519 (B_1g_), and 639 cm^-1 ^(E_g_) [26]. From the Raman spectra, it is evident that both the undoped and Eu-doped TiO_2 _powders are in an anatase phase. There appeared no apparent impurity-related modes in the Raman spectra of doped samples, in agreement with the obtained XRD results. In order to appreciate the differences between the spectra more clearly, the position and the full width at half maximum [FWHM] of the E_g _mode at 144 cm^-1 ^are also presented in Table 3. With the increase of doping concentration, the position of the Raman bands, in particular the E_g _mode near 144 cm^-1^, shifts towards higher wavenumbers and their intensities decrease drastically. The observation can be attributed to the reduction of particle size in the Eu-doped samples. When the grain size decreases to the nanometer scale, the vibrational properties of materials are influenced greatly. Mainly, a volume contraction occurs within the nanoparticles due to the size-induced radial pressure, which leads to an increase in the force constants because of the decrease in the interatomic distances. In vibrational transitions, the wavenumber varies approximately in proportion to *k*^1/2^, where *k *is the force constant. Consequently, the Raman bands shift towards a higher wavenumber due to the increasing force constants [27]. The sudden reduction in scattering intensity, particularly of the E_g _mode, may be due to the breakdown of long-range translational crystal symmetry caused by the incorporated defects.

**Table 3:** The position and FWHM of the E_g _mode in the undoped and Eu-doped TiO_2 _nanoparticles

Sample	Position of the E_g _mode (cm^-1^)	FWHM (cm^-1^)
TiO_2_:Eu 0.0%	144.2	10.22
TiO_2_:Eu 0.5%	146.7	14.67
TiO_2_:Eu 1.0%	146.3	12.63
TiO_2_:Eu 2.5%	146.09	13.24
TiO_2_:Eu 5.0%	147.6	13.82

FWHM, full width at half maximum.

**Figure 6:**
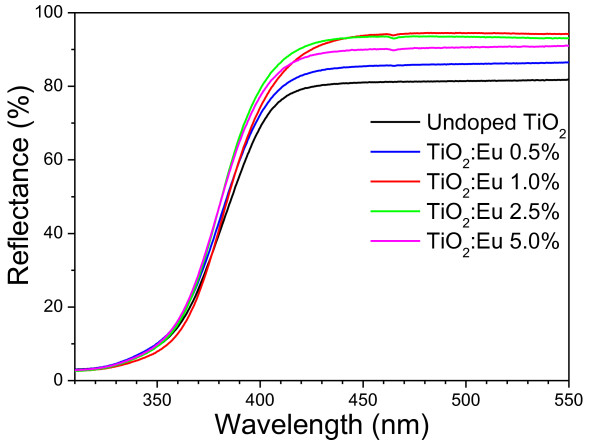
**UV-Vis diffuse reflectance spectra for the undoped and Eu-doped TiO_2 _phosphor nanoparticles**.

**Figure 7:**
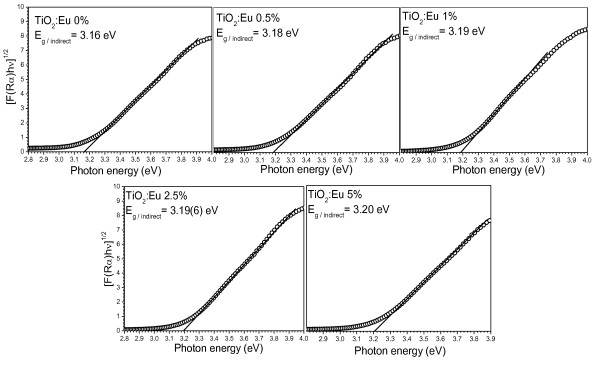
**Kubelka-Munk plots and bandgap energy estimation of pure and Eu-doped TiO_2 _nanoparticles for indirect transition**.

Spectroscopic measurement of diffuse reflectance in UV-Vis spectral range is a standard technique for the determination of the bandgap of powder samples [28]. Figure 6 shows the diffuse reflectance spectra of the undoped and Eu-doped titania particles after thermal treatment. A sharp decrease in reflectance started at about 415 nm for the undoped TiO_2 _samples due to strong absorption. On increasing the incorporated Eu content, the absorption edge suffered a gradual blueshift. The reflectance spectra were analyzed using the Kubelka-Munk relation to convert the reflectance into a Kubelka-Munk function (equivalent to the absorption coefficient), *F (R*_α_), using the relation:

(2) $$F\left(R_{\alpha }\right)\;=\;{\left(1\;-R_{\alpha }\right)}^{2}∕2R_{\alpha },$$

where *R*_α _is the reflectance of an infinitely thick sample with respect to a reference at each wavelength. Bandgap energies of the samples were estimated from the variation of the Kubelka-Munk function with photon energy. Figure 7 presents the Kubelka-Munk plots for the undoped and Eu-doped samples used to determine their bandgap energy associated with an indirect transition. It can be observed that the indirect bandgap increases gradually with the increase of doping concentration. However, the estimated indirect bandgap values (3.16 to 3.20 eV) for all the samples were very close to the reported indirect bandgap value of anatase [29]. With the increase of incorporated Eu content, the bandgap energy of the TiO_2 _nanostructures increased systematically. This behavior is very similar to the previously reported results [30], where the authors observed a blueshift in the bandgap of Eu-doped CdS nanorods with the increase of doping concentration. The reason of such bandgap energy increment has been proposed as the gradual movement of the conduction band of TiO_2 _above the first excited state of Eu^+3 ^due to the increased dopant incorporation. Incorporated Eu^+3 ^ions at the first excited state interact with the electrons of the conduction band of TiO_2_, resulting in a higher energy transfer from the TiO_2 _to Eu^+3 ^ions. However, an increased absorption in the visible range and red shift of the energy bandgap have been observed by Yu et al. on doping TiO_2 _nanotubes with Fe^+3 ^ions [31]. Such an opposite behavior has been explained through the creation of dopant levels near the valence band of TiO_2 _on Fe^+3 ^ion incorporation. Therefore, the relative shift of the absorption edge of the semiconductor depends strongly on the difference between the ionic radius of the dopant and the host cations, as well as on the chemical nature of the dopants.

**Figure 8:**
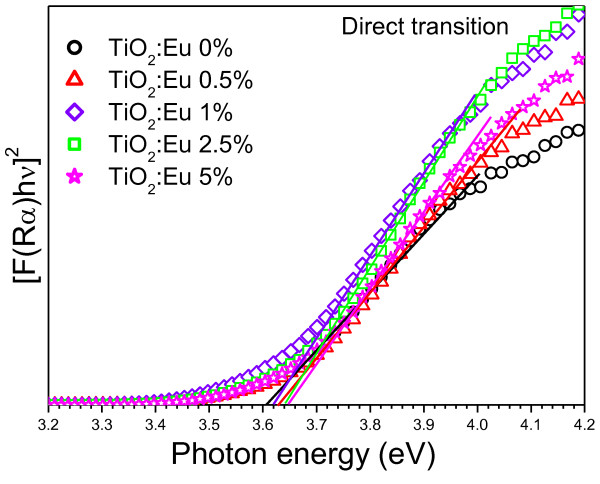
**Kubelka-Munk-transformed diffuse reflectance spectra of the Eu-doped nanoparticles used for the estimation of direct bandgap**.

To evaluate the bandgap energy of the nanoparticles associated to their direct transition, [*F(R*_α_)hv]^2 ^vs. hv were plotted (Figure 8). The estimated bandgap values (obtained from linear fits of the square of the remission function) are quite larger than those associated with indirect transitions which has been reported previously [32].

**Figure 9:**
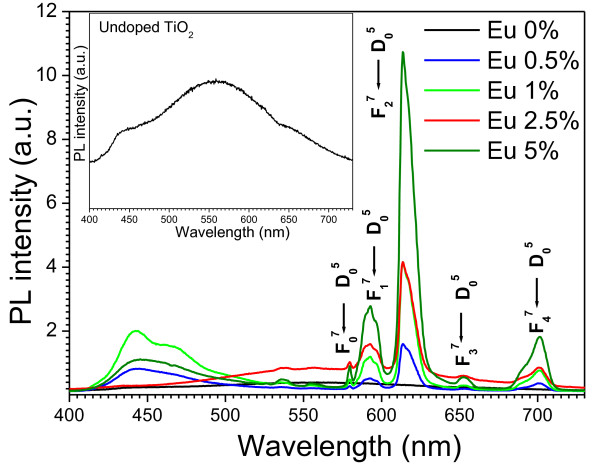
**Room-temperature PL spectra of the undoped and Eu-doped titania nanoparticles before thermal annealing**.

**Figure 10:**
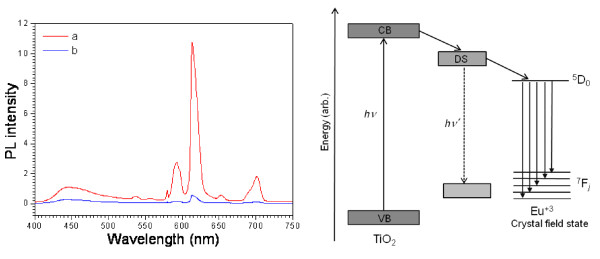
**PL spectra of the 5.0% Eu-doped titania nanoparticles**. (**a**) Before and (**b**) after thermal annealing (left). Schematic illustration of the possible mechanism of energy transfer from the TiO_2 _host to Eu^+3 ^(right). VB, CB, and DS correspond to the valence band, conduction band, and defect state, respectively.

Figure 9 shows the PL spectra of the undoped and Eu-doped titania nanoparticles before thermal treatment. Eu^+3^-doped phosphor nanoparticles show several sharp and well-resolved emission lines associated with Eu^+3 ^ions which correspond to radiative relaxations from the ^5^*D*_0 _level to its low-lying multiplets ^7^*F_j_*. The strongest emission centered at around 612 nm corresponds to the electrical dipole transition (^5^*D*_0 _- ^7^*F_2_*) of Eu^+3 ^ions which give the red color in the luminescence signals. In the literature, it has been reported that this transition is possible only if Eu^+3 ^ions occupy a site without an inverse symmetry [33]. Other emission peaks centered around 578, 592, 651, and 700 nm are associated with ^5^*D*_0 _- ^7^*F_0_*, ^5^*D*_0 _- ^7^*F_1 _*(magnetic dipole transition), ^5^*D*_0 _- ^7^*F_3_*, and ^5^*D*_0 _- ^7^*F_4 _*transitions of Eu^+3 ^ions, respectively. With the increase of Eu^+3 ^content from 0.5 to 5 mol% (nominal), the PL intensity increases systematically. Besides the characteristic emission peaks attributed to the Eu^+3 ^ions, we can also find a broad emission band in between 415 and 530 nm for the Eu-doped samples. In the case of 0.5%, 1%, and 5.0% doped samples, the band is centered at around 442 nm along with a small shoulder at 466 nm for 1% Eu-doped titania nanoparticles. Commonly, PL emission of anatase TiO_2 _is attributed to three different physical origins: self-trapped excitons, oxygen vacancies, and surface states (defect) [34]. The 442-nm band most probably originated from the self-trapped excitons localized on TiO_6 _octahedra [35], whereas the 466-nm band is attributed to oxygen vacancies [36]. It is interesting to note that for the 2.5% Eu-doped sample, the blue emission (emission in between 415 and 530 nm) has been decreased drastically, indicating that the relative intensity of the red and blue emissions can be tailored by adjusting the concentration of dopant ions in the TiO_2 _lattice. The undoped TiO_2 _sample revealed a broad low-intensity band centered at 560 nm with a small shoulder at higher energy (440 nm; inset of Figure 7). This visible luminescence band arises from the radiative recombination of electrons via intrinsic surface states of TiO_2 _nanoparticles [37]. It is well known that in case of nanoparticles, surfaces play important roles as the surface-to-volume ratio becomes increasingly large at a nanometer size. As TiO_2 _is a strongly ionic metal oxide, the filled valance band is mainly composed of the outermost 2*p *orbitals of oxygen atoms, and the lowest conduction band is derived from titanium 3*d *orbitals. When some titanium atoms are exposed to the surface of nanoparticles, they get oxidized into Ti^+3^, Ti^+2^, or Ti^+ ^oxidation states, and localized energy levels are introduced within the forbidden gap [38]. These intrinsic surface states act as luminescence centers under an appropriate excitation as can be seen in the present work. Figure 10 shows the PL spectra recorded at room temperature for the 5.0 mol% Eu-doped titania nanoparticles before and after thermal treatment. For the unannealed Eu-doped samples, the narrow emission peaks are clearly attributed to f-f transitions of Eu^+3 ^ions. However, the PL spectrum of the heat-treated sample did not reveal the characteristic emission peaks of Eu^+3 ^ions except the ^5^*D*_0 _- ^7^*F_2 _*transition of a very low intensity and the visible luminescence band corresponding to anatase TiO_2 _nanostructures. Similar observations have also been reported in the literature [39]. In the as-grown (unannealed) samples, the amorphous TiO_2 _matrix not only acts as a good host for well-dispersed Eu^+3 ^ions, but also functions as a good sensitizer by transferring the absorbed energy to Eu^+3 ^ions [40]. Electrons are initially excited to the conduction band of TiO_2 _on irradiating UV light and then relaxed to the defect states. Since the defect states of TiO_2 _are located at higher energies than those of the emitting state (^5^D_0_) of Eu^+3 ^ions, energy transfer to the crystal field states (^7^F*_j_*) of Eu^+3 ^occurs, resulting in efficient PL [41]. This energy transfer process is schematically depicted in Figure 10 at the right. When the sample is annealed at 500°C, all the PL emissions almost disappeared (Figure 10). This could be related to the transformation of amorphous titania to a fully crystalline anatase phase which presents a higher density, making more difficult for Eu^+3 ^ions to locate at the site of Ti^+4 ^due to the large difference in their ionic radii [42]. Thus, the well-dispersed Eu^+3 ^ions in the unannealed amorphous titania tend to be segregated outwards. This might cause the undesired Eu-Eu interactions acting as a luminescent quencher and leads to a drastic decrease in the PL intensity.

## Conclusion

In conclusion, highly uniform, spherical-shaped Eu-doped TiO_2 _phosphor particles could be synthesized through a simple sol-gel technique at a large scale. The low-cost phosphor particles are about 50 nm in average diameter and have about 10% size dispersion. With the increasing nominal doping concentration up to 5.0 mol%, the average diameter of the particles reduces to 38 nm. Under ultraviolet excitation, the phosphor particles show the characteristic emission corresponding to the *^5^D_0 _*- *^7^F_j _*transition of Eu^+3 ^ions along with a broad band in the 400- to 500-nm range belonging to anatase TiO_2_. Thermal annealing-induced crystallization of the nanoparticles causes a drastic reduction of PL emission intensity, suggesting amorphous TiO_2 _as an ideal framework for an efficient energy transfer between the titania host and incorporated Eu^+3 ^ions. The low fabrication cost, high yield, controlled morphology, and good luminescent performance of the as-grown TiO_2_:Eu^+3 ^nanoparticles provide the possibility of using them as efficient red-emitting phosphors.

## Competing interests

The authors declare that they have no competing interests.

## Authors' contributions

MP proposed the original idea, carried out most of the experimental works associated with synthesis and characterization of the samples, analyzed the results, and prepared the manuscript. UP improved the original idea, helped in analyzing the results, and revised the manuscript. JMGyJ performed the PL measurements and analyzed the data. FP-R designed and coordinated the whole work. All authors read and approved the final manuscript.
